# Enhanced antimicrobial efficacy and energy efficiency of low irradiance 405-nm light for bacterial decontamination

**DOI:** 10.1007/s00203-024-03999-1

**Published:** 2024-05-23

**Authors:** Lucy G. Sinclair, John G. Anderson, Scott J. MacGregor, Michelle Maclean

**Affiliations:** 1 The Robertson Trust Laboratory for Electronic Sterilisation Technologies (ROLEST), Department of Electronic & Electrical Engineering, University of Strathclyde, Glasgow, UK; 2 Department of Biomedical Engineering, University of Strathclyde, Glasgow, UK

**Keywords:** 405 nm light, Violet-blue light, Antimicrobial photoinactivation, ESKAPE pathogens, Antibacterial, Low irradiance

## Abstract

Due to its increased safety over ultraviolet light, there is interest in the development of antimicrobial violet-blue light technologies for infection control applications. To ensure compatibility with exposed materials and tissue, the light irradiances and dose regimes used must be suitable for the target application. This study investigates the antimicrobial dose responses and germicidal efficiency of 405 nm violet-blue light when applied at a range of irradiance levels, for inactivation of surface-seeded and suspended bacteria. Bacteria were seeded onto agar surfaces (10^1^–10^8^ CFUplate^−1^) or suspended in PBS (10^3^–10^9^ CFUmL^−1^) and exposed to increasing doses of 405-nm light (≤ 288 Jcm^−2^) using various irradiances (0.5–150 mWcm^−2^), with susceptibility at equivalent light doses compared. Bacterial reductions ≥ 96% were demonstrated in all cases for lower irradiance (≤ 5 mWcm^−2^) exposures. Comparisons indicated, on a per unit dose basis, that significantly lower doses were required for significant reductions of all species when exposed at lower irradiances: 3–30 Jcm^−2^/0.5 mWcm^−2^ compared to 9–75 Jcm^−2^/50 mWcm^−2^ for low cell density (10^2^ CFUplate^−1^) surface exposures and 22.5 Jcm^−2^/5 mWcm^−2^ compared to 67.5 Jcm^−2^/150 mWcm^−2^ for low density (10^3^ CFUmL^−1^) liquid exposures (P ≤ 0.05). Similar patterns were observed at higher densities, excluding *S. aureus* exposed at 10^9^ CFUmL^−1^, suggesting bacterial density at predictable levels has minimal influence on decontamination efficacy. This study provides fundamental evidence of the greater energy efficacy of 405-nm light for inactivation of clinically-significant pathogens when lower irradiances are employed, further supporting its relevance for practical decontamination applications.

**Supplementary Information:**

The online version contains supplementary material available at 10.1007/s00203-024-03999-1.

## Introduction

The emergence of multi-drug resistant (MDR) bacteria is considered one of the greatest public health threats of the modern day. In efforts to drive and facilitate research and development of novel antimicrobial therapeutics, the World Health Organization (WHO) recently published a list of global MDR ‘priority’ pathogens which pose the greatest threat to human health (WHO 2017). Therein, the ESKAPE pathogens (*Enterococcus faecium*, *Staphylococcus aureus*, *Klebsiella pneumoniae*, *Acinetobacter baumannii*, *Pseudomonas aeruginosa* and *Enterobacter cloacae*), which collectively represent the leading cause of nosocomial infections worldwide (Santajit and Indrawattana 2016), were appointed high and critical priority status (WHO 2017). The development of novel therapeutics and disinfection technologies to enhance prevention of MDR bacterial infections, particularly those induced by ESKAPE pathogens, is thus of significant research interest at present.

One such approach is the use of antimicrobial violet-blue light, with wavelengths within the region of 400–420 nm. Exposure to these wavelengths induces the photoexcitation of endogenous porphyrin molecules within microbial cells, and a cascade of cellular processes resulting in the localised production of reactive oxygen species causing oxidative damage and cell death (Hamblin et al. 2005; Maclean et al. 2008). Although less germicidal than UV-light, the technology is inherently antimicrobial, and can achieve this at exposure levels compatible with mammalian cells (Ramakrishnan et al. 2014; Tomb et al. 2018), and because of this, there has been interest in its development for infection control applications which involve human exposure or treatment of sensitive materials, such as environmental decontamination applications (Maclean et al. 2010; 2013; Bache et al. 2012, 2018; Murrell et al. 2019; Sinclair et al. 2023a, b) or wound treatment (McDonald et al. 2011; Dai et al. 2013a, b).

To ensure compatibility in such studies where sensitive materials or tissue is exposed, light irradiances of ~ 0.3–20 mWcm^−2^ have generally been used. This can differ from studies solely investigating the fundamental antimicrobial properties of violet-blue light, which can use considerably higher light irradiances (up to ~ 200 mWcm^−2^) (Hamblin et al. 2005; Guffey and Wilborn 2006; Murdoch et al. 2012; McKenzie et al. 2014; Tomb et al. 2014; Moorhead et al. 2016).

Importantly, there is currently little evidence to understand the inactivation efficacy, on a per unit dose basis, of violet-blue light when employed using low versus high irradiance light sources—an important consideration towards practical application of the technology. Accordingly, the present study aimed to expand knowledge about the germicidal efficiency (GE) of low irradiance versus high irradiance 405-nm light, on a per unit energy basis. Experiments investigated the inactivation of ESKAPE pathogens at various population densities, both on surfaces and in liquid suspension, and the GE at different irradiance levels was compared in order to determine the impact of bacterial load and illumination intensity on 405-nm light inactivation efficacy.

## Materials and methods

### Bacterial preparation

The bacterial strains used in this study were: *Enterococcus faecium* LMG 11423, Staphylococcus aureus NCTC 4135, *Klebsiella pneumoniae* LMG 3081, *Acinetobacter baumannii* LMG 1041, *Pseudomonas aeruginosa* LMG 9009 and *Enterobacter cloacae* LMG 2783 (LMG: Laboratorium voor Microbiologie, Universiteit Gent, Belgium; NCTC: National Collection of Type Cultures, Colindale, UK). Bacteria were cultured in 100 mL nutrient broth (Oxoid Ltd, UK), with the exception of *E. faecium* and *A. baumannii* which were cultured in tryptone soya broth (Oxoid Ltd, UK), at 37 °C under rotary conditions (120 rpm) for 18–24 h (C24 Incubator Shaker, New Brunswick Scientific, USA). Following cultivation, broths were centrifuged at 3939 × g for 10 min (Heraeus Labofuge 400R; Kendro Laboratory Products, UK), and the cell pellet was resuspended and serially diluted in phosphate buffered saline (PBS; Oxoid Ltd, UK) to obtain the required population density, in colony forming units per millimetre (CFUmL^−1^), for experimental use. Population densities were checked by cultivation on nutrient agar plates (NA; Oxoid Ltd, UK), with the exception of *A. baumannii* and *E. faecium*, which were cultivated on tryptone soya agar (TSA; Oxoid Ltd, UK).

### 405-nm light sources

**Fig. 1:**
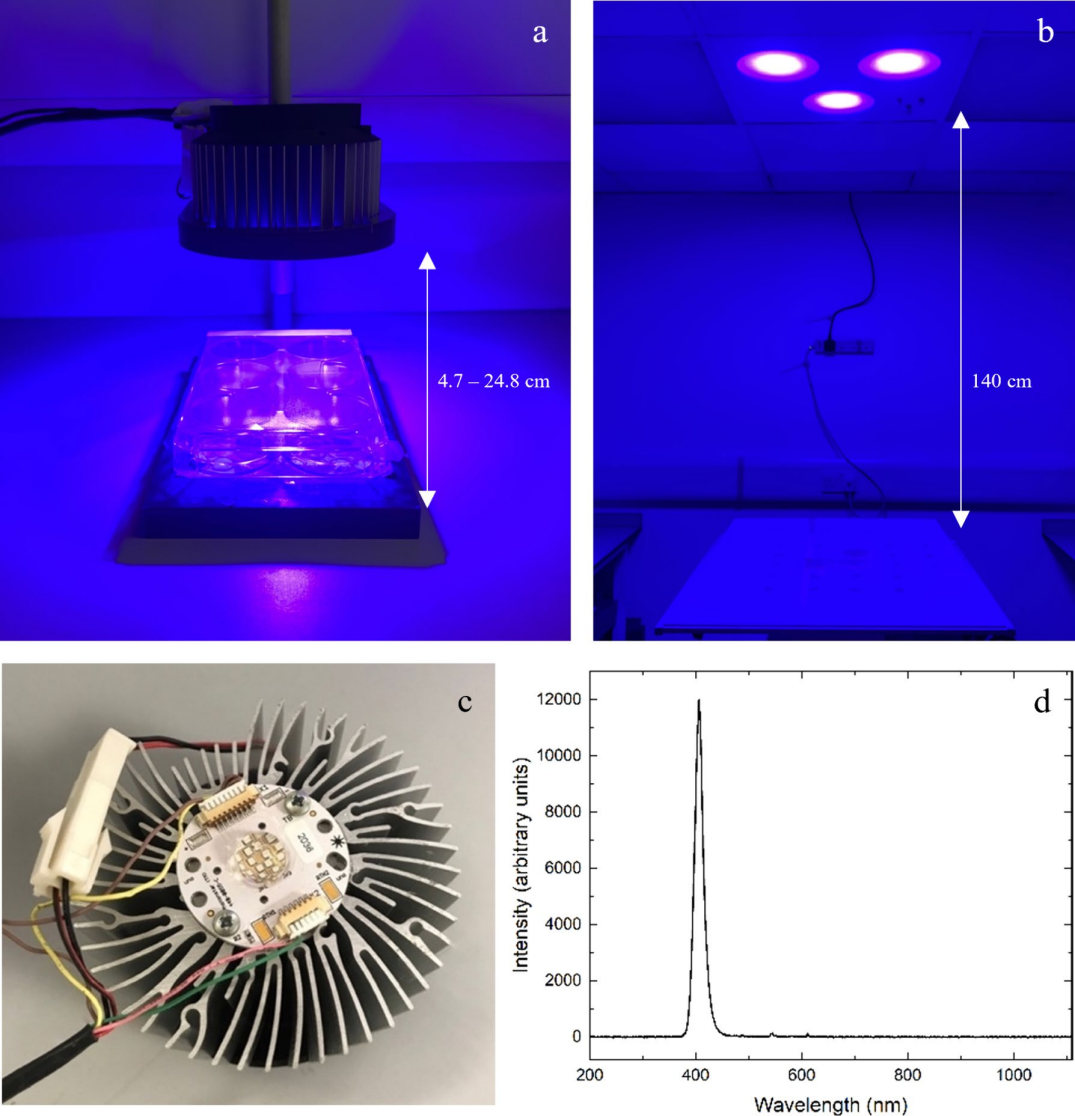
Light sources for exposures of ESKAPE pathogens: **a** small-scale bench top system, **b** ceiling-mounted system, **c** 405-nm LED array and **d** emission spectra of the 405-nm light output of the array. All emission spectra data was captured using an HR4000 spectrometer (Ocean Optics, Germany) and Spectra Suite software version 2.0.151

Two light sources were used for experimental testing: a small-scale benchtop system which provided irradiances between 5 and 150 mWcm^−2^ (Fig. 1a), and a ceiling-mounted system used for irradiances of 0.5 mWcm^−2^ (Fig. 1b). Both systems utilised 405-nm light emitting diode (LED) arrays (ENFIS PhotonStar Innovate UNO 24, PhotonStar Technologies Ltd, UK—Fig. 1c), which were powered by a 62 V LED driver (Philips, Netherlands) and delivered light at a peak output of approximately 405-nm with a bandwidth of 16-nm at full-width half maximum (FWHM; Fig. 1d). The benchtop system was comprised of a single LED array mounted on a polyvinyl chloride (PVC) housing which held the array above a base plate on which the bacterial samples were positioned (Fig. 1b). To fix the irradiance level used for sample exposure, the distance between the array and the sample was adjusted, and the irradiance was measured using a radiant optical power meter and a photodiode detector (Oriel Instruments): 5 mWcm^−2^–24.8 cm; 10 mWcm^−2^–17.5 cm; 50 mWcm^−2^–8.0 cm; 100 mWcm^−2^–5.7 cm; 150 mWcm^−2^–4.7 cm.

The ceiling-mounted 405-nm LED system (University of Strathclyde, UK; Patent Number: US8398264B2) was positioned 1.4 m above a worktop on which samples were exposed (Fig. 1b). All bacterial samples were positioned directly underneath the light source on this surface, which provided 405-nm light irradiances of 0.5 ± 0.03 mWcm^−2^ across each sample.

### 405-nm light exposure of surface-seeded bacteria

#### Influence of irradiance levels on surface-seeded bacterial inactivation

To establish if the irradiance level used influences the inactivation kinetics of surface-seeded bacteria, inactivation kinetics of each ESKAPE pathogen was established for exposures to equivalent doses at irradiance levels of 0.5–50 mWcm^−2^. Bacteria were seeded and spread onto 50 mm agar plates using an L-shaped spreader (either NA or TSA, depending on the organisms) at a density of 10^2^ CFUplate^−1^; selected to represent the typical upper levels of contamination reported on surfaces within hospital isolation rooms (Maclean et al. 2013), Once dried, the seeded plates with lids off (n = 3) were then exposed to increasing durations of light treatment at three irradiance levels: 0.5, 5 and 50 mWcm^−2^. The exposure times used for each irradiance level were selected in order to ensure equivalent light doses (3–180 Jcm^−2^) were delivered to the samples; calculated using Eq. 1:

1 $$Dose \left(J {cm}^{-2}\right)=Irradiance \left(W {cm}^{-2}\right)\times Exposure\, Time (s)$$

Following light treatment, the lids were replaced on the plates and sample plates were incubated at 37 °C for 18–24 h (IP 250 Incubator; LTE Scientific, UK) and surviving bacterial colonies were enumerated and recorded as CFUplate^−1^, with results compared to control samples held under ambient room lighting conditions.

#### Influence of population density on surface-seeded bacterial inactivation

To establish if the density of surface-seeded bacteria influences the inactivation kinetics when exposed to low irradiance 0.5 mWcm^−2^ 405-nm light, bacteria were seeded and spread onto 50 mm agar plates using an L-shaped spreader (NA or TSA, depending on the organism) at densities of 10^1^, 10^2^, 10^3^, 10^4^, 10^5^, 10^6^, 10^7^ and 10^8^ CFUplate^−1^ and, once dried (approximately taking 1 min), exposed to 0.5 mWcm^−2^ 405-nm light with the lids off for 16 h (28.8 Jcm^−2^) and 24 h (43.2 Jcm^−2^). Following light treatment, the lids were replaced on the plates, and the plates were incubated at 37 °C for 18–24 h. Post-incubation, plates were photographed for qualitative analysis, with results compared to control samples held under ambient room lighting conditions. Negligible temperature increases were recorded on illuminated sample surfaces throughout light exposures.

### 405-nm light exposure of liquid-suspended bacteria

#### Influence of irradiance levels on liquid-suspended bacterial inactivation

To establish the influence of irradiance on the efficacy of 405-nm light for inactivation of liquid-suspended bacteria *S. aureus* and *P. aeruginosa*, selected as a representative Gram-positive and Gram-negative species, respectively, were exposed to increasing durations of light treatment at five irradiance levels: 5, 10, 50, 100 and 150 mWcm^−2^. Bacterial suspensions were prepared at a population density of 10^3^ CFUmL^−1^, to represent low-level contamination and enable subsequent enumeration, and 250 µL volumes (n = 3) were held in a 96-well plate (providing a sample depth of 7.8 mm) covered by an adhesive plate seal (Thermo Scientific, UK), to prevent evaporation, and positioned below the light source. To ensure any light adsorption by the adhesive plate seal was accounted for, irradiance measurements were taken through the material, and the height of the light source was adjusted accordingly to ensure the desired irradiance reached bacterial samples. The exposure times used for each irradiance level were selected in order to ensure equivalent light doses (22.5–180 Jcm^−2^) were delivered to the samples [Eq. 1].

Post-treatment, 100 µL samples (n = 2) were spread plated onto agar plates and incubated at 37 °C for 18–24 h. Post-incubation, colonies were enumerated with results reported as surviving bacterial load in CFUmL^−1^, and compared to control samples which were exposed to ambient room lighting.

#### Influence of population density on liquid-suspended bacterial inactivation

To establish if the inactivation kinetics of liquid-suspended bacteria upon 405-nm light exposure is influenced by changes in bacterial density, *S. aureus* and *P. aeruginosa* were exposed at population densities of 10^3^, 10^5^, 10^7^ and 10^9^ CFUmL^−1^. 3 mL volumes (n = 3) were transferred to a 6-well plate, providing a sample depth of 3.2 mm, and exposed to increasing doses of 405-nm light at irradiances of 5, 50 and 150 mWcm^−2^, with the exposure times selected to ensure equivalent light doses (36–288 Jcm^−2^) were delivered to the samples [Eq. 1]. All 6-well plates were covered with an adhesive plate seal to prevent evaporation, and so irradiance measurements were taken through the material as described in Sect. “Influence of Irradiance Levels on Liquid-Suspended Bacterial Inactivation” to ensure the desired irradiance reached bacterial samples. Post-treatment, 100 µL samples (n = 2) were plated onto agar plates and incubated at 37 °C for 18–24 h. Post-incubation, colonies were enumerated with results reported as surviving bacterial load in CFUmL^−1^, and compared to control samples which were exposed to ambient room lighting.

### Data and statistical analysis

Experimental data points constitute the mean of triplicate biological replicates (n = 3), with an additional two technical replicates (n = 6) in the case of liquid-suspended bacterial samples. Error bars represent the standard deviation (SD) of these values. Data was analysed using two sample t-tests and one-way ANOVA with Tukey post-hoc test on Minitab Statistical Software Release 19 (Minitab Ltd, UK) with significant differences identified at the 95% confidence interval (P ≤ 0.05). Quantitative data is presented as either the percentage surviving bacterial population in comparison to non-exposed equivalent control populations, bacterial counts in log_10_ CFUmL^−1^ (for tests using higher population density suspensions), or as GE values. GE is defined as the log_10_ reduction of a bacterial population by inactivation per unit dose in Jcm^−2^ (Maclean et al. 2009) and was calculated by log_10_(N/N_0_), where N_0_ and N represent bacterial populations prior and post exposure, respectively, divided by the applied dose, in Jcm^−2^, required to achieve ≥ 95% inactivation. For analysis of low-density bacterial populations exposed on surfaces (10^2^ CFUplate^−1^) and in liquid suspension (10^3^ CFUmL^−1^), complete/near-complete inactivation was considered as ≥ 95% reductions in 405-nm light exposed populations in comparison to non-exposed equivalent control populations. In certain instances, counted bacterial samples were below the limit of detection (LOD; 10 CFUmL^−1^); however, this data has been included to demonstrate the complete or near-complete inactivation effect achieved.

## Results

### Enhanced efficacy of lower irradiance exposure for surface-seeded inactivation

**Fig. 2:**
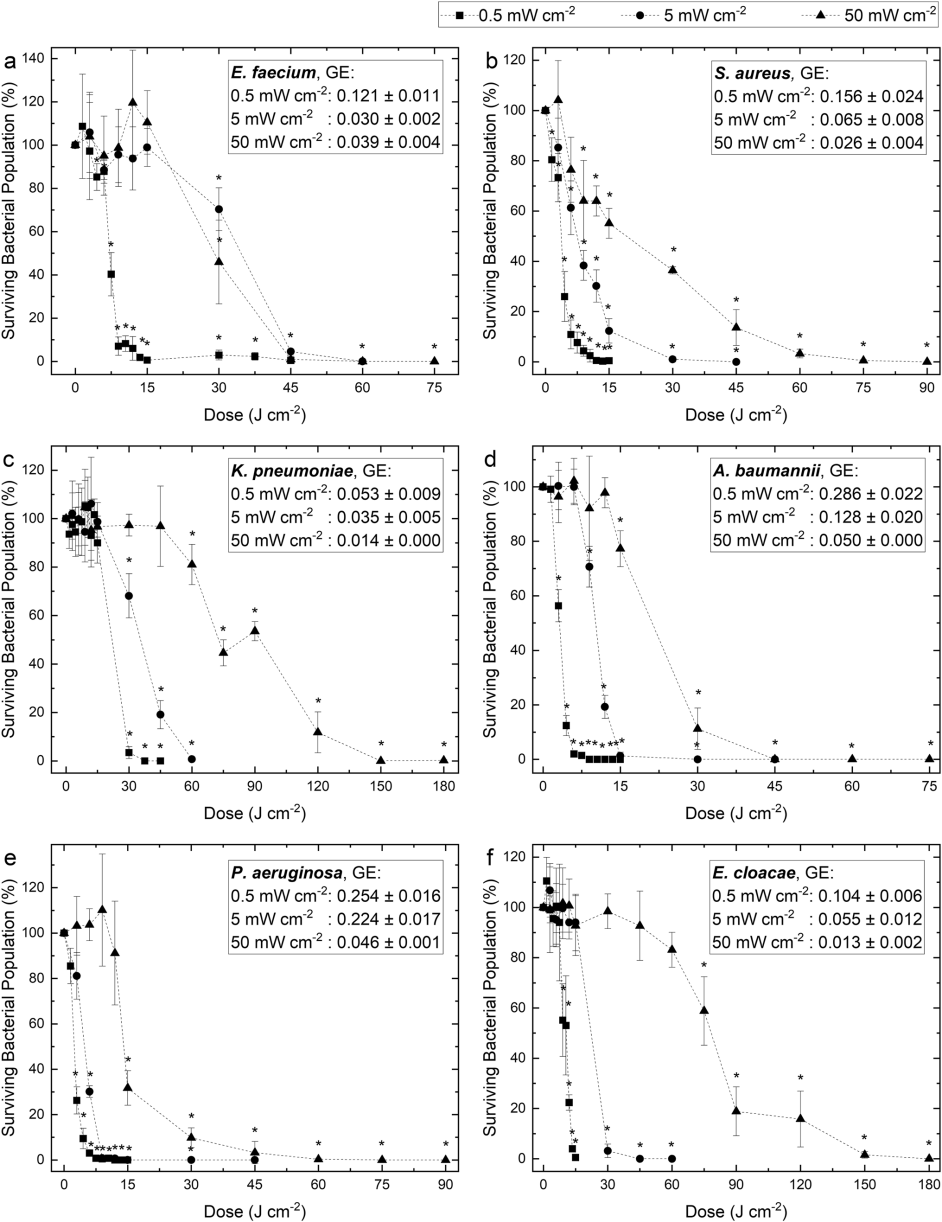
Inactivation of surface-seeded (~ 10^2^ CFUplate^-1^) ESKAPE pathogens, **a** *E. faecium*, **b** *S. aureus*, **c** *K. pneumoniae*, **d** *A. baumannii*, **e** *P. aeruginosa* and **f** *E. cloacae*, upon exposure to increasing doses of 405-nm light at irradiances of 0.5, 5 and 50 mWcm^−2^. Surviving bacterial populations are presented as percentages with respect to equivalent non-exposed control populations. In all instances, GE was calculated at the dose at which complete or near complete (≥ 95%) inactivation was achieved. Each data point represents the mean value ± SD (n = 3). Asterisks (*) represent data points in which there was a significant reduction in the surviving bacterial population in comparison to the equivalent non-exposed control population (P ≤ 0.05)

Inactivation kinetics of ESKAPE bacteria (10^2^ CFUplate^−1^) following exposure to an increasing dose of antimicrobial 405-nm visible light at three independent irradiance regimes are presented in Fig. 2. Results demonstrate a significant downward trend in the surviving bacterial population for all organisms tested when light dose was increased, and non-exposed control samples showed no significant change throughout (P > 0.05). Although each species displayed varying susceptibility to exposure, inactivation efficacy (on a per unit dose basis) was significantly enhanced for all organisms when the dose was delivered at lower irradiance.

Exposed to the highest irradiance of 50 mWcm^−2^, significant reductions of ESKAPE pathogens compared to non-exposed control populations (19–68.3% reductions; P < 0.05) were collectively demonstrated following exposure to doses of 9–75 Jcm^−2^ (3–25 min). At this same irradiance, doses of 45–150 Jcm^−2^ (15–50 min) were required to achieve complete or near-complete (≥ 96.73%) inactivation. When exposed at a lower irradiance of 5 mWcm^−2^, significant reductions of all species (29.3–69.9% reductions; P < 0.05) was demonstrated following exposure to lower doses of 6–30 Jcm^−2^ (20 min–1 h 40 min). Complete or near-complete inactivation (≥ 95.47%) was achieved by all species at this same irradiance following exposure to 15–60 Jcm^−2^ (50 min–3 h 20 min). When exposed to the lowest irradiance of 0.5 mWcm^−2^, significant reductions in ESKAPE pathogens (14.8–96.6% reductions; P < 0.05) was demonstrated following exposure to just 3–30 Jcm^−2^ (1 h 40 min–16 h 40 min). At this same irradiance, 6–30 Jcm^−2^ (3 h 20 min–16 h 40 min) was required to achieve complete or near-complete (≥ 96%) inactivation. Comprehensively, the dose required for a 1 log_10_ reduction was significantly less for all species when exposed using 0.5 mWcm^−2^ (6–30 Jcm^−2^) in comparison to both 5 mWcm^−2^ (9–60 Jcm^−2^) and 50 mWcm^−2^ (30–150 Jcm^−2^).

In all cases, GE values for exposures to 0.5 mWcm^−2^ were significantly greater (P ≤ 0.05) than GE values for exposures to both 5 and 50 mWcm^−2^ (Fig. 2). The greatest difference was demonstrated by *S. aureus*, whereby GE for near-complete (≥ 95.6%) inactivation was 6 times greater when exposed using 0.5 mWcm^−2^ compared to 50 mWcm^−2^ (0.156 vs 0.026, respectively).

There were significant differences in the susceptibility of the various ESKAPE pathogens to 405-nm light inactivation at each application of irradiance. Exposed at the lowest irradiance of 0.5 mWcm^−2^, *S. aureus*, *A. baumannii* and *P. aeruginosa* proved to be most susceptible to treatment, collectively requiring ≤ 9 Jcm^−2^ to achieve complete or near complete inactivation. Exposed at the highest irradiance application of 50 mWcm^−2^, results indicate *E. faecium*, *A. baumannii* and *P. aeruginosa* were the most susceptible to inactivation, requiring 45 Jcm^−2^ for complete/ near complete inactivation, whereas *K. pneumoniae* and *E. cloacae*, which were demonstrated to be the least susceptible, each required much greater doses of 150 Jcm^−2^ to achieve similar reductions.

### Efficacy of lower irradiance exposure for surface-seeded inactivation at varying densities

**Fig. 3:**
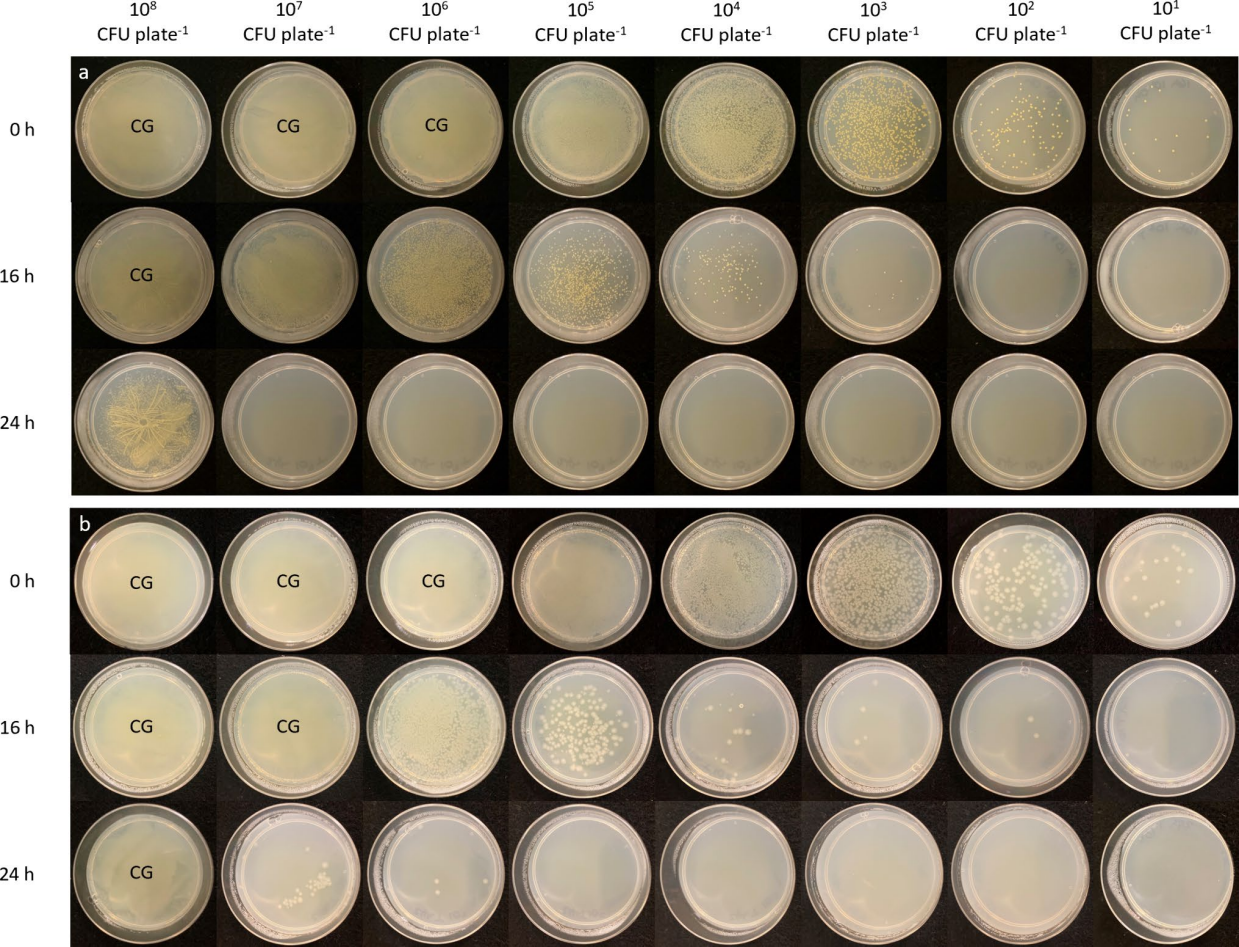
Appearance of **a** *S. aureus* and **b** *P. aeruginosa* at 10^8^–10^1^ CFUplate^-1^, after exposure to 0.5 mWcm^-2^ 405-nm light for 16 h and 24 h (28.8 and 43.4 Jcm^-2^, respectively). CG represents plates with confluent growth of bacteria

To qualitatively investigate the influence of population density on bacterial inactivation using lower irradiance 405-nm light exposure, bacteria at population densities of 10^1^–10^8^ CFUplate^−1^ were exposed to 0.5 mWcm^−2^ for 16 h (28.8 Jcm^−2^) and 24 h (43.4 Jcm^−2^), with this irradiance selected due to its improved GE (Fig. 2). Figure 3 displays agar plates seeded with *S. aureus* and *P. aeruginosa*, chosen as representative Gram-positive and Gram-negative species, respectively. Results for the other bacterial species are shown in Online Resource 1.

Visual observations indicate that lower irradiance 405-nm light is capable of inactivating surface-seeded bacteria at a range of initial population densities (Fig. 3, Online Resource 1). Similar reductions were demonstrated at each initial seeding density as the light dose was increased, with results indicating that all bacteria presented at high surface population densities can be reduced by lower irradiance 405-nm light exposure as the duration of exposure, and thus applied dose, is increased. By comparison of the two representative species presented in Fig. 3, *S. aureus* appears to demonstrate greater susceptibility than that of *P. aeruginosa*, with 24 h exposure resulting in complete elimination of 10^7^ CFUplate^−1^ *S. aureus* populations in comparison to just 10^5^ CFUplate^−1^ *P. aeruginosa* populations. Nevertheless, results overall demonstrate successful inactivation of all ESKAPE pathogens, even at high density levels (Fig. 3, Online Resource 1).

### Enhanced efficacy of lower irradiance exposure for liquid-suspended inactivation

**Fig. 4:**
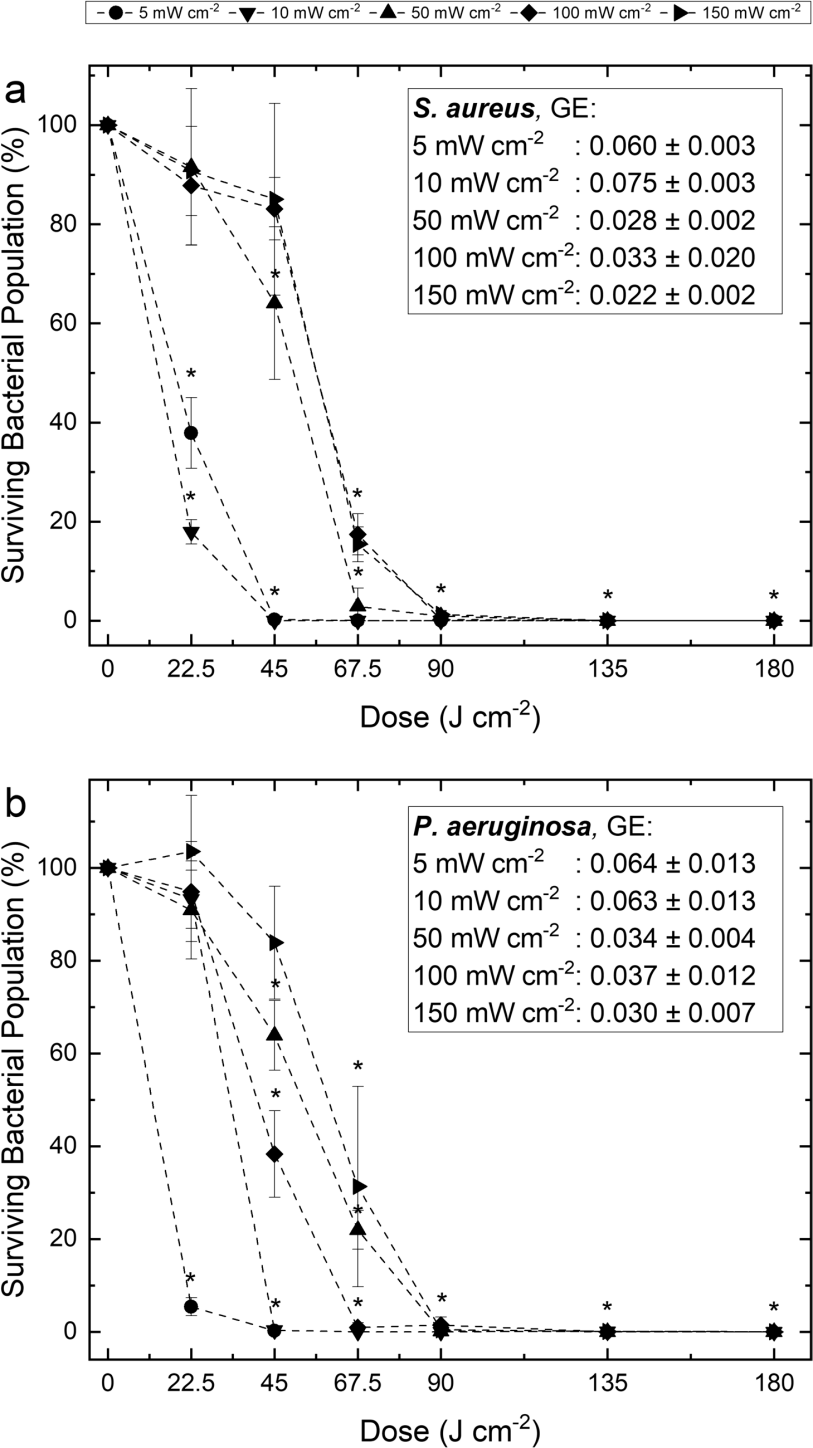
Inactivation of **a** *S. aureus* and **b** *P. aeruginosa* suspended in PBS upon exposure to 405-nm light up to a dose of 180 Jcm^−2^ at irradiances of 5, 10, 50, 100 and 150 mWcm^−2^. Surviving bacterial populations are presented as percentages with respect to equivalent non-exposed controls. In all instances, GE was calculated at the dose at which complete or near-complete (≥ 95%) inactivation was achieved. Each data point represents the mean value ± SD (n = 6); LOD = 10 CFUmL^−1^. Asterisks (*) represent data points in which there was a significant reduction in the surviving bacterial population in comparison to the equivalent non-exposed control population (P ≤ 0.05)

Figure 4 presents the inactivation kinetics of liquid-suspended *S. aureus* and *P. aeruginosa* (10^3^ CFUmL^−1^) exposed to increasing doses of 405-nm light at five independent irradiances. In all cases, a downward trend in surviving bacterial populations was achieved when the light dose was increased, and no significant changes were observed in non-exposed control populations throughout treatment (P ≤ 0.05). At all irradiances, complete/near-complete inactivation (> 98.6%) of both species was achieved following exposure to 90 Jcm^−2^, however, general trends indicate an increased susceptibility to inactivation when exposed using lower irradiance: for both species, GE values for exposures to ≤ 10 mWcm^−2^ were significantly greater (P ≤ 0.05) than GE values for exposures to ≥ 50 mWcm^−2^ (Fig. 4).

*S*. *aureus* (Fig. 4a) demonstrated increased susceptibility when lower irradiance light was employed. Following 45 Jcm^−2^, reductions of 99.7% (2.94 log_10_) and 100% (3.37 log_10_) were observed for *S. aureus* exposed at 5 and 10 mWcm^−2^, respectively; in comparison to just 35.9% (0.21 log_10_), 16.9% (0.08 log_10_) and 15.0% (0.08 log_10_) reductions when exposed at 50, 100 and 150 mWcm^−2^, respectively. The energy required to achieve complete/near-complete (≥ 99.7%) *S. aureus* inactivation was significantly lower when exposed at lower irradiances (P ≤ 0.05): 45 Jcm^−2^ using 5 and 10 mWcm^−2^ compared to double this energy (90 Jcm^−2^) using 50, 100 and 150 mWcm^−2^.

*P*. *aeruginosa* similarly demonstrated increased susceptibility to inactivation when exposed at lower irradiance (Fig. 4b), with significantly greater inactivation at 45 Jcm^−2^, 99.7% (2.86 log_10_) and 99.6% (2.81 log_10_) reductions were observed using 5 and 10 mWcm^−2^, respectively; in comparison to just 36.1% (1.25 log_10_), 61.7% (0.43 log_10_) and 16.1% (0.08 log_10_) reductions using 50, 100 and 150 mWcm^−2^, respectively. Complete/near-complete (≥ 99.1%) *P. aeruginosa* inactivation was achieved using up to two times less dose at lower irradiance in comparison to higher irradiances: 45 Jcm^−2^ was required for 5 and 10 mWcm^−2^ exposures, in comparison to 67.5 Jcm^−2^ for 100 mWcm^−2^ exposures and 90 Jcm^−2^ for both 50 and 150 mWcm^−2^ exposures.

### Efficacy of lower irradiance exposure for liquid-suspended inactivation at varying densities

**Fig. 5:**
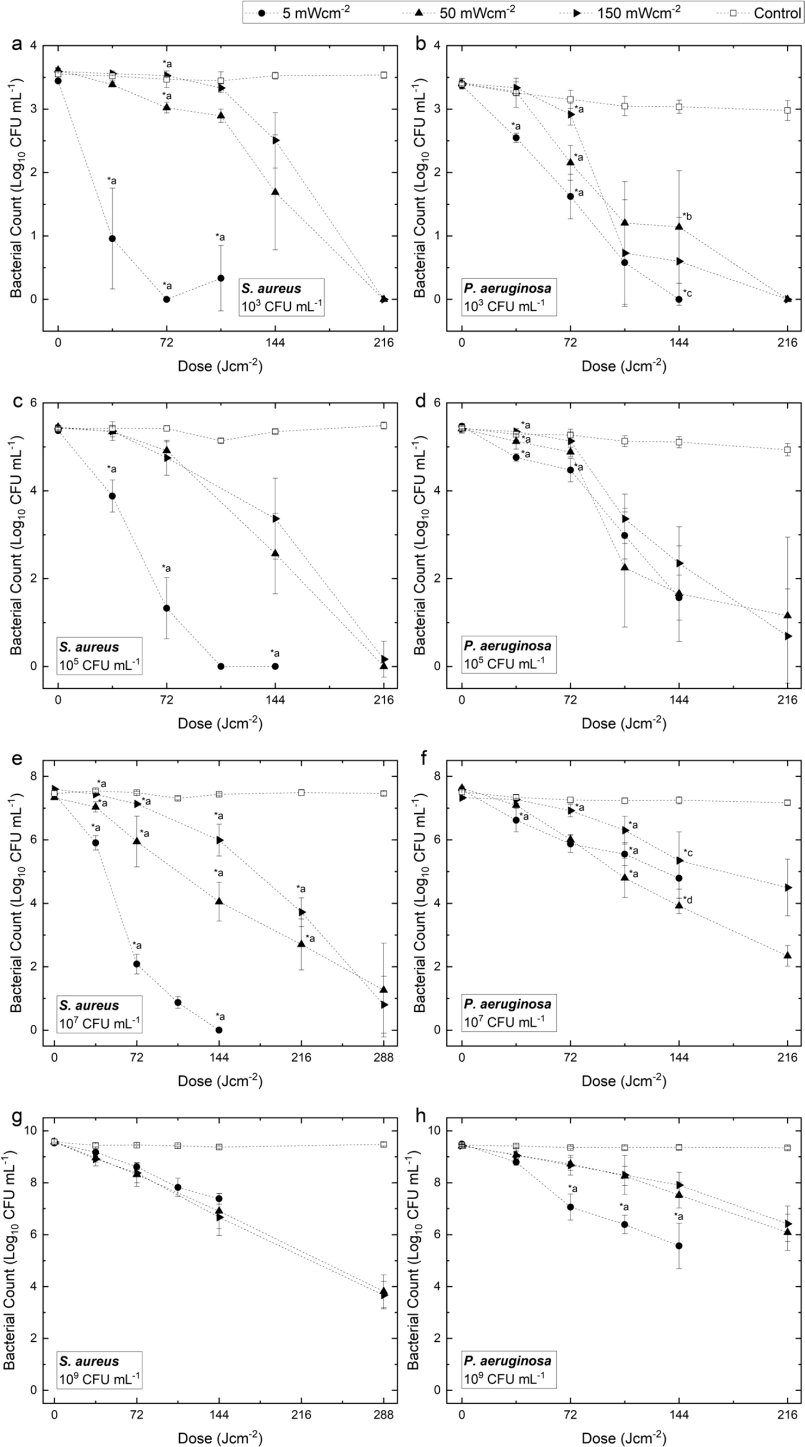
Inactivation kinetics of *S. aureus* suspended in PBS at initial population densities of **a** 10^3^ CFUmL^−1^, **c** 10^5^ CFUmL^−1^, **e** 10^7^ CFUmL^−1^ and **g** 10^9^ CFUmL^−1^ and *P. aeruginosa* at initial population densities of **b** 10^3^ CFUmL^−1^, **d** 10^5^ CFUmL^−1^, **f** 10^7^ CFUmL^−1^ and (h) 10^9^ CFUmL^−1^ upon exposure to increasing doses of 405-nm light at irradiances of 5, 50 and 150 mWcm^−2^. Surviving bacterial populations are presented in log_10_ CFUmL^−1^. Each data point represents the mean value ± SD (n = 3); LOD = 10 CFUmL^−1^. Asterisks (*) represents levels of inactivation significantly different to other irradiances at a particular applied dose (P ≤ 0.05): *a, significantly different to all other irradiances; *b, significantly different to 150 mWcm^−2^; *c, significantly different to 5 mWcm^−2^

The inactivation kinetics of liquid-suspended *S. aureus* and *P. aeruginosa*, at initial population densities of 10^3^, 10^5^, 10^7^ and 10^9^ CFUmL^−1^, upon exposure to increasing doses of 405-nm visible light at three independent irradiances are presented in Fig. 5. Due to the higher cell densities investigated in this section, bacterial counts are reported as log_10_ CFUmL^−1^ rather than as the percentage of surviving bacteria in comparison to equivalent controls.

The inactivation efficiency of *S. aureus* upon 405-nm light exposure was shown to be significantly enhanced when lower irradiances (5 mWcm^−2^) were employed at population densities of ≤ 10^7^ CFUmL^−1^ and, conversely, when higher irradiances were employed (150 mWcm^−2^) at a population density of 10^9^ CFUmL^−1^ (P ≤ 0.05). At 10^3^ CFUmL^−1^ (Fig. 5a), log_10_ bacterial counts were significantly lower upon exposure to 5 mWcm^−2^ compared to both 50 and 150 mWcm^−2^ (P < 0.001) at all light doses measured. Approximately 4–6 times less dose was required to achieve ≥ 96.4% inactivation when exposed at the lowest irradiance: 36 Jcm^−2^ at 5 mWcm^−2^ (2.46 log_10_ CFUmL^−1^ reduction) compared to 144 Jcm^−2^ at 50 mWcm^−2^ (1.93 log_10_ CFUmL^−1^ reduction) and 216 Jcm^−2^ at 150 mWcm^−2^ (3.59 log_10_ CFUmL^−1^ reduction). Similar patterns were observed at 10^5^ CFUmL^−1^ (Fig. 5c) and 10^7^ CFUmL^−1^ (Fig. 5e). Discordantly, when exposed at 10^9^ CFUmL^−1^ (Fig. 5g), higher irradiances achieved significantly greater reductions per unit dose: 36 Jcm^−2^ achieved a 0.66 log_10_ CFUmL^−1^ (72.9%) reduction using 150 mWcm^−2^, compared to a 0.61 log_10_ CFUmL^−1^ (55.6%) reduction using 50 mWcm^−2^ and a 0.36 log_10_ CFUmL^−1^ (48.0%) reduction using 5 mWcm^−2^. At this density, the dose required for a 1 log_10_ reduction (Fig. 5g) was 1.5 times greater using 5 mWcm^−2^ compared to 150 mWcm^−2^.

The inactivation efficacy of *P. aeruginosa* upon 405-nm light exposure was shown to be significantly enhanced when lower irradiance exposures (5 mWcm^−2^) were employed at all population densities (P ≤ 0.05). At 10^3^ CFUmL^−1^ (Fig. 5b), 36 Jcm^−2^ resulted in a 0.82 log_10_ CFUmL^−1^ (59.8%) reduction using 5 mWcm^−2^, which was significantly greater than the 0.13 log_10_ CFUmL^−1^ (27.8%) reduction achieved using 50 mWcm^−2^ (P ≤ 0.05) and the 0.07 log_10_ CFUmL^−1^ (11.02%) reduction achieved using 150 mWcm^−2^ (P ≤ 0.05). Similar results were observed using 10^5^ CFUmL^−1^ (Fig. 5d) and 10^7^ CFUmL^−1^ (Fig. 5f): with the latter demonstrating that two times less dose was required for ≥ 97.3% inactivation (≥ 2.03 log_10_ CFUmL^−1^ reductions) when exposed using the two lower irradiances (108 Jcm^−2^) compared to the highest (216 Jcm^−2^). In contrast to *S. aureus*, inactivation of 10^9^ CFUmL^−1^ populations (Fig. 5h) was significantly greater at the lowest irradiance (5 mWcm^−2^) compared to the two higher irradiances up to a dose of 108 Jcm^−2^ (P ≤ 0.05). In addition, 2–3 times less dose was required for ≥ 98.0% inactivation when exposed at 5 mWcm^−2^ (72 Jcm^−2^; 2.43 log_10_ CFUmL^−1^) in comparison to that of 50 mWcm^−2^ (144 Jcm^−2^; 1.90 log_10_ CFUmL^−1^) and 150 mWcm^−2^ (216 Jcm^−2^; 3.02 log_10_ CFUmL^−1^). Overall, the dose required for a 1 log_10_ reduction of 10^3^ and 10^5^ CFUmL^−1^ populations (Fig. 5b and d) was the same for all irradiance applications (108 Jcm^−2^; P > 0.05); and at 10^7^ and 10^9^ CFUmL^−1^, the dose required for a 1 log_10_ reduction (Fig. 5f and h) was 2 times lower when exposed using 5 mWcm^−2^ compared to 150 mWcm^−2^.

Table 1 presents the GE of liquid-suspended bacteria for each irradiance as initial bacterial populations are increased. For *S. aureus*, GE values for inactivation of initial densities of 10^3^–10^7^ CFUmL^−1^ were shown to be significantly greater when the lowest employed irradiance of 5 mWcm^−2^ was used in comparison to that of higher irradiance exposures (P ≤ 0.001). At the highest bacterial density (10^9^ CFUmL^−1^), however, GE values for exposure to this lowest irradiance of 5 mWcm^−2^ was significantly lower than that of both higher irradiance exposures (P = 0.001). For *P. aeruginosa*, GE values for each irradiance application are comparable, with greater values presented at lower irradiances for 10^5^–10^9^ CFUmL^−1^ populations.

**Table 1:** Germicidal efficiencies for ≥ 95% inactivation of liquid-suspended *S. aureus* and *P. aeruginosa* (10^3^–10^9^ CFUmL^−1^; 3 mL volumes) upon exposure to identical doses of 405-nm light using irradiances ranging from 5 to 150 mWcm^−2^

Population density (CFUmL^−1^)	*S. aureus*			*P. aeruginosa*		
	5 mWcm^−2^	50 mWcm^−2^	150 mWcm^−2^	5 mWcm^−2^	50 mWcm^−2^	150 mWcm^−2^
10^3^	0.068 ± 0.021*	0.013 ± 0.006	0.017 ± 0.000	0.022 ± 0.006	0.019 ± 0.006	0.021 ± 0.008
10^5^	0.054 ± 0.009*	0.038 ± 0.000	0.037 ± 0.003	0.020 ± 0.004	0.027 ± 0.012*	0.017 ± 0.005
10^7^	0.043 ± 0.006*	0.033 ± 0.004*	0.026 ± 0.003*	0.020 ± 0.004	0.022 ± 0.006	0.013 ± 0.005*
10^9^	0.015 ± 0.003*	0.018 ± 0.005	0.019 ± 0.001	0.032 ± 0.007*	0.013 ± 0.003	0.013 ± 0.003

Each data point represents the mean value ± SD (n = 3). For each data point, GE was calculated at the dose at which ≥ 95% inactivation was achieved. Asterisks (*) represent irradiance exposures which had a significantly different GE value from that of all other irradiance exposures (P ≤ 0.05)

The temperature of liquid samples was also monitored for all treatments. The greatest temperature increases were observed with the longest exposures at highest irradiance (10^9^ CFUmL^−1^; 150 mWcm^−2^; ≥ 24 min; 42 ℃). Despite this increase, regimes applied using lower irradiances were still found to achieve greater inactivation on a per unit dose basis, indicating that temperature had minimal effect.

## Discussion

This study has successfully demonstrated the efficacy of lower irradiances of 405-nm light for the inactivation of key nosocomial bacteria presented at various population densities on surfaces and in liquid suspension, and has additionally highlighted the enhanced inactivation efficacy of bacteria when exposed to a fixed dose of 405-nm light using lower irradiance levels (≤ 5 mWcm^−2^) compared to higher irradiance levels (≥ 50 mWcm^−2^).

As mentioned, given its increased safety profile over ultraviolet light, there is interest in the development of antimicrobial violet-blue light for infection control applications which require exposure of sensitive materials or occupied environments, and to ensure compatibility with such applications, lower irradiance light is generally used. For environmental decontamination purposes, violet-blue light is typically employed at levels of ~ 0.5 mWcm^−2^ to enable safe continuous use in occupied environments (Maclean et al. 2010), and so this particular irradiance was selected as a representative. Results in Figs. 2 and 3 indicate that all surface-exposed ESKAPE pathogens (10^1^–10^8^ CFUplate^−1^) were reduced by exposure to 0.5 mWcm^−2^, with greater levels of inactivation achieved as light dose was increased. When exposed at 10^2^ CFUplate^−1^ (Fig. 2), which was chosen to represent the typical upper levels of contamination reported within hospital isolation rooms (Maclean et al. 2013), the susceptibility of each ESKAPE pathogen was variable, however, complete/near-complete (≥ 96.6%) inactivation of each species individually was achieved using 3–30 Jcm^−2^ (3.3–16.6 h). Comparative exposures of liquid-suspended bacteria to 0.5 mWcm^−2^ could not be conducted due to the limited viability of bacteria whilst suspended for lengthy periods in PBS. Bacteria will not likely survive for extended periods without nutrition, and given that multiple hours would likely be required to achieve significant bacterial inactivation when exposed in PBS to 0.5 mWcm^−2^, this irradiance level was not included in these instances. Alternatively, an irradiance level of 5 mWcm^−2^ was selected, with this irradiance being in the region of those suggested for wound disinfection applications (5–20 mWcm^−2^) (McDonald et al. 2011; Dai et al. 2013a, b). Use of 5 mWcm^−2^ was shown to successfully reduce populations of liquid-suspended *S. aureus* and *P. aeruginosa*, selected as Gram-positive and Gram-negative representatives, respectively, for exposures in liquid, with near-complete (≥ 99.7%) inactivation achieved for both species upon exposure to a dose of 45 Jcm^−2^ (2.5 h; Fig. 4).

In addition to comparing the bactericidal efficacy of different doses of 405-nm light, this study has also provided novel information on comparative bactericidal efficacy when the same dose of light is applied at different irradiation levels. The results provide key evidence to demonstrate the enhanced bactericidal efficacy of lower irradiance 405-nm light in comparison to that of higher irradiance exposures, per unit dose. Results in Figs. 2 and 6 indicate all surface-seeded ESKAPE bacteria (10^2^ CFUplate^−1^) required a significantly (P < 0.05) lower dose to achieve complete/near-complete (≥ 95.47%) inactivation, and exhibited significantly higher GE for inactivation (P < 0.05) when exposed using lower irradiance levels: exposures to 0.5 mWcm^−2^ required up to two times less energy than that required by exposures to 5 mWcm^−2^, and up to five times less energy than that required by exposures to 50 mWcm^−2^. Similarly, results in Figs. 4 and 7 suggest liquid-suspended *S. aureus* and *P. aeruginosa* also required lower doses to achieve significant levels of inactivation, and the GE for inactivation was significantly greater, when exposed using lower irradiance levels: both species required 1.5–2 times greater energy when exposed at high-irradiance (≥ 50 mWcm^−2^) compared to low-irradiance (≤ 10 mWcm^−2^) (P ≤ 0.05).

Interestingly, these findings can be considered to conflict with previous studies which depicted a dose-dependency associated with 405-nm light exposure; suggesting inactivation increases with applied dose, irrespective of the light delivery regime (high intensity/short duration or low intensity/long duration) (Endarko et al. 2012; Murdoch et al. 2012; Barneck et al. 2016). It is important to consider, however, that these studies compared exposures to irradiances within the same order of magnitude, and thus it is possible that light intensity may have a greater influence on inactivation efficacy when irradiances of varying orders of magnitude are compared. Murdoch et al., for example, exposed suspensions of *Listeria monocytogenes* to 108 Jcm^−2^ of 405-nm light using irradiances of 10–30 mWcm^−2^ and found, although the log_10_ reduction achieved was slightly greater at lower intensity, there was no significant difference in the level of inactivation achieved at the different irradiance exposures (Murdoch et al. 2012). Similarly, Barneck et al. investigated equivalent 405-nm light dose exposures using irradiances of 2.38–2.89 and 8.85–9.71 mWcm^−2^ on surface-seeded *S. aureus*, S*treptococcus pneumoniae*, *Escherichia coli* and *P. aeruginosa* and demonstrated similar log_10_ reductions for both equivalent radiant exposures (Barneck et al. 2016). The authors did note, however, that from the appearance of growth distribution patterns on treated culture plates, low intensity/long duration exposure regimes may provide a superior bactericidal effect due to the continuous nature of treatment (Barneck et al. 2016).

The concept that differences in inactivation efficacy may become increasingly evident when comparing irradiance applications of greater orders of magnitude is further supported by Endarko et al., who found no significant difference (P > 0.05) in the 405-nm light inactivation rate of *L. monocytogenes* when exposed to a constant dose using irradiances ranging from 44 to 85.6 mWcm^−2^ but did demonstrate significantly greater inactivation when exposed to a constant dose using 8.6 mWcm^−2^ in comparison to 85.6 mWcm^−2^ (Endarko et al. 2012). More recently, Sinclair et al. successfully inactivated bacteriophage phi6, as a surrogate for SARS-CoV-2, using a 405-nm light EDS at irradiances similar to that employed in this study (0.5 mWcm^−2^) and demonstrated up to 5.8 times greater log_10_ reductions with up to 28-fold greater GE using this light source in comparison to a higher 50 mWcm^−2^ light source (Sinclair et al. 2023b). Although the results of this study cannot be directly compared with a viral surrogate, it is of interest to note that the enhancement in inactivation demonstrated here is likely apparent for a broad-spectrum of microbial species.

These findings provide key evidence to indicate the application of 405-nm light dose has an important role in the mechanisms of microbial inactivation. The improvement in bactericidal efficacy demonstrated at lower irradiance exposures may be due to specific levels of energy required for microbial inactivation, and it is possible that bacteria may adopt different response mechanisms when exposed to high intensity/short duration regimes in comparison to low intensity/long duration regimes. It has previously been identified that, when light irradiance is increased, there is the potential for photoinactivation to be limited by saturation of the electronic absorption of porphyrins (Maclean et al. 2016) or a depletion in oxygen supplies (Moseley et al. 2006; Rogers 2012), which could result in a surplus of excited porphyrin molecules which are unable to participate in the photoinactivation process. However, in this study, the light irradiances used were substantially lower than the irradiance levels which could instigate saturation of the electronic absorption of porphyrins, and the inactivation kinetics of *S. aureus* at an initial density of 10^9^ CFUmL^−1^ shown in Fig. 5 indicate that oxygen is not lacking at the higher irradiances employed in this study. A possible explanation for the observed effects could be the result of bacterial responses to a relatively gentle oxidative stress for a prolonged period of time, as opposed to a higher oxidative stress for a much shorter period of time. Whilst much is known about the damaging effects of ROS on microbial cells and on ROS defence mechanisms, there is relatively little understanding of how the interplay between these factors affects microbial populations in different environments (Imlay 2019). It is known that high ROS levels can cause genomic mutations and particularly RNA damage (Seixas et al. 2022) as well as inducing oxidative modification of proteins, lipids and glycans which can lead to metabolic malfunction and cell death. Conversely, low-level local ROS play an important role both as redox-signalling molecules in a wide spectrum of pathways involved in the maintenance of cellular homeostasis and regulating key transcription factors (Schieber and Chandel 2014; Fasnacht and Polacek 2021).

Clearly ROS can have diverse effects on microbial cells and the results of the present study, demonstrating that greater GE occurred when 405 nm radiation was exposed at a relatively low irradiance level over an extended period compared with a higher irradiance over a shorter period, further adds to this body of knowledge. A plausible explanation is that such low-level exposure over a prolonged period is in some way more damaging to the cell but alternatively it could be that ROS cellular defences are less effective under these conditions or perhaps as is also possible that the study results reflect some combination of enhanced lethality and impaired defence associated with prolonged exposure to low level 405 nm light. It is also important for future study to consider the minimum threshold irradiance level for antimicrobial activity. It is likely that, below a certain level, any ROS produced as a result of these low-level light exposures are within levels capable of being detoxified by bacterial antioxidant defence mechanisms. Going forward it will be of interest to investigate the mechanism accountable for these differing microbial responses, as a better understanding of this fundamental concept could assist in the optimisation of low power, energy efficient antimicrobial lighting systems.

When exposed on surfaces, the qualitative data in Fig. 4 indicates that lower irradiance (0.5 mWcm^−2^) 405-nm light could inactivate increasingly high bacterial population densities as the time of exposure, and thus applied dose, was increased. It is important to highlight, however, that bacteria exposed to nutritional stressors prior to UV-A light treatment, such as heat, pH or chemicals, have previously been shown to develop cross-protection against optical damage, primarily due to the induction of starvation-induced proteins which can protect and provoke repair mechanisms (Fernádez and Pizarro 1999). Although this effect has yet to be demonstrated for violet-blue visible light exposure, it is important to consider the potential protective influence of environmental stressors within practical environments. Nutritional agar surfaces were chosen as the exposure medium in this study as a means to provide highly reproducible inactivation kinetics within the laboratory setting; however, prior studies have demonstrated the efficacy of low irradiance 405-nm light systems in reducing clinical environment contamination, where environmental stressors were likely present (Maclean et al. 2010, 2013; Bache et al. 2012, 2018; Murrell et al. 2019; Amodeo et al. 2023). The demonstration of successful bacterial inactivation using lower irradiance 405-nm light on nutritious media and at population densities which significantly exceed that expected of contaminant levels within healthcare settings further supports the suitability of this technology for environmental decontamination. Low irradiance 405-nm light systems are designed to be used continuously within healthcare environments such that, rather than providing a quick and complete disinfection, consistently low levels of contamination can be maintained as bacterial levels fluctuate with room activity.

In terms of the comparative susceptibility of ESKAPE pathogens, results in Fig. 2 indicate *A. baumannii*, *S. aureus* and *P. aeruginosa* were most susceptible to 0.5 mWcm^−2^; collectively requiring doses ≤ 6 Jcm^−2^ to achieve 1 log_10_ reductions, in comparison to *E. cloacae*, *E. faecium* and *K. pneumoniae*, which required doses of 13.5, 9 and > 15 Jcm^−2^, respectively. These results reflect those of Hoenes et al., who similarly found that *A. baumannii*, *S. aureus* and *P. aeruginosa* required lower doses of 405-nm light than that of other ESKAPE pathogens to achieve a 1 log_10_ reduction (Hoenes et al. 2021). The authors exposed ESKAPE pathogens at a higher irradiance (20 mWcm^−2^) than employed in this study (Hoenes et al. 2021), suggesting the comparative susceptibility of each species to treatment is independent of light irradiance application. This is corroborated by exposures to 50 mWcm^−2^ shown in Fig. 2, which demonstrated similar patterns of susceptibility. These differences are likely due to differences in both the distribution and quantity of endogenous porphyrins produced by different bacterial cells (Nitzan et al. 2004; Maclean et al. 2009; Kumar et al. 2015). Regardless, complete/near-complete (≥ 96.6%) inactivation of all species was demonstrated using low irradiance 405-nm light, similar to that employed for whole-room decontamination, within realistic exposure times for system use (16.6 h). These findings support those of Sinclair et al., who similarly demonstrated that 0.5 mWcm^−2^ 405-nm light could successfully inactivate a range of nosocomial and foodborne pathogens, with complete reductions of each species demonstrated in 2–16 h (Sinclair et al. 2023a).

Similar to the results reported above for surface exposed bacteria, this study has also demonstrated the energy effectiveness of lower irradiance 405-nm light when bacterial populations are treated in liquid suspension. Results in Figs. 5 and 7 demonstrate that the enhancement in inactivation efficacy observed for lower irradiance exposures is apparent for various *S. aureus* and *P. aeruginosa* populations in suspension. These findings support those of Zhang et al. (2023), who, whilst investigating the energy efficiency of pulsed 405-nm light for inactivation of *E. coli* suspensions, demonstrated that exposure time has greater influence over inactivation than that of irradiance, thus suggesting that greater reductions could be achieved when lower irradiances are employed over extended time periods, compared to higher irradiances over shorter time periods. By contrast, however, results for *S. aureus* exposed at an initial population density of 10^9^ CFUmL^−1^ deviate from the overall findings of this study for reasons that are not yet fully understood. As previously discussed, this observation is likely due to differences in the species-specific response of bacteria to relatively gentle oxidative stress for prolonged time periods, compared to higher oxidative stress for shorter time periods, however, further investigation is necessary to fully elucidate the mechanism for these differing microbial responses. The current study provides evidence regarding the fundamentals of 405-nm light inactivation of liquid-suspended bacteria; however, it is important that future studies address its efficacy under conditions representative of those used for clinical applications.

Structural differences between Gram-positive and Gram-negative bacteria cells may play a role in the efficacy of light transmission through the exposed sample, thus affecting the efficacy of 405-nm light inactivation demonstrated in this study. Gram-positive bacteria, which lack an outer cell membrane, contain surrounding layers of peptidoglycan which are substantially thicker than those surrounding Gram-negative cells (Silhavy et al. 2010). It is therefore possible that this layer may increase 405-nm light attenuation, meaning the inactivation of Gram-positive bacteria is limited as cell density increases. Other possible factors such as any cellular production of light absorbing pigments, e.g. *S. aureus* carotenoids, may also affect light penetration into dense populations. Further, at the higher cell densities investigated in this study, it is likely that bacterial clumping will result in shading effects which will likely influence light penetration through suspensions. These hypotheses align with a recent systemised review of current findings regarding microbial inactivation by violet-blue light exposure, which demonstrated a slight increase in the dose requirements for a 1 log_10_ reduction in bacteria upon increasing population density (Tomb et al. 2018). Further, Bumah et al. (2013) previously demonstrated that the bactericidal effect is not influenced by population density; however, reduced light penetration through suspensions will be limiting. Although contamination is unlikely to be presented at these higher densities, this study sought to examine how bacterial density, and thus shading, may affect 405-nm light inactivation. It is important to note however, only one Gram-positive and Gram-negative representative species were investigated here, and so further study into the comparative interactions and resulting damage mechanisms of 405-nm light between Gram-positive and Gram-negative bacteria are essential to further this understanding.

The findings of this study provide evidence to demonstrate the bactericidal efficacy of lower irradiance 405-nm light for the inactivation of nosocomial pathogens which, in conjunction with its established safety benefits, highly supports use of this technology for infection control applications involving the exposure of occupied environments, tissue or sensitive materials. Further investigation into the associated photochemical inactivation mechanisms involved in 405-nm light inactivation of bacterial cells, and the response of mammalian cells to such exposures, is required to establish the biochemical reasons for the increased efficiency of lower irradiance 405-nm light application and better characterise the safety profile of the technology. Further, for practical application, additional knowledge about how GE may be affected by practical challenges, such as the presence of biofilms, biological fluids, or organic material will be important. An enhanced understanding of this field of study will be critical to optimise the use of lower irradiance 405-nm light and augment clinical translatability of this technology for widespread implementation across the infection control sector.

## Conclusions

Overall, this study has successfully demonstrated the broad-spectrum antimicrobial efficacy and enhanced GE of lower irradiances of 405-nm light for the treatment of a panel of key nosocomial pathogens. Significant inactivation of ESKAPE pathogens was achieved at a range of bacterial cell densities with ≥ 96% inactivation demonstrated for lower irradiance (≤ 5 mWcm^−2^) 405-nm light exposures. Comparisons indicated, on a per unit dose basis, significantly lower doses were required to achieve significant reductions of all surface-seeded species when exposed at lower irradiances. Similar findings showing better efficiency with lower irradiances were also apparent when bacteria were in suspension. This study provides fundamental evidence of the susceptibility of ESKAPE pathogens to lower irradiance 405-nm light exposure, further supporting its use for infection control applications which involve human exposure or treatment of sensitive materials.

### Supplementary Information

Below is the link to the electronic supplementary material.

Supplementary file1 (PDF 373 KB)

## Data Availability

Data supporting this publication are stored by the University of Strathclyde. Details of the data and how it can be accessed are available from the University of Strathclyde KnowledgeBase at https://doi.org/10.15129/22c276f9-b3f1-4e59-a470-6ed7c34d530a.
