# Supplementary material for: Enhanced antimicrobial efficacy and energy efficiency of low irradiance 405-nm light for bacterial decontamination
Source: Arch Microbiol. 2024 May 23;206(6):276. doi: 10.1007/s00203-024-03999-1 (PMC11111507; doi:10.1007/s00203-024-03999-1)
Supplement: Supplementary file 1 — Supplementary file1 (PDF 373 KB) [file 203_2024_3999_MOESM1_ESM.pdf]

Enhanced Antimicrobial Efficacy and Energy Efficiency of Low Irradiance 405-nm Light for Bacterial Decontamination

Archives of Microbiology

Lucy G Sinclair[1] (0009-0007-8121-7795), lucy.g.sinclair@strath.ac.uk

John G Anderson[1] (0000-0003-4151-1619), j.g.anderson@strath.ac.uk

Scott J. MacGregor[1] (0000-0002-0808-585X), scott.macgregor@strath.ac.uk

Michelle Maclean[1,2*] (0000-0001-5750-0397), michelle.maclean@strath.ac.uk

[1]The Robertson Trust Laboratory for Electronic Sterilisation Technologies (ROLEST), Department of Electronic & Electrical Engineering, University of Strathclyde, Glasgow, UK, [2]Department of Biomedical Engineering, University of Strathclyde, Glasgow, UK.

*Corresponding author email: michelle.maclean@strath.ac.uk

Online Resource 1

This file contains the inactivation kinetics of *E. faecium* (Fig. 1), *K. pneumoniae* (Fig. 2), *A. baumannii* (Fig. 3) and *E. cloacae* (Fig. 4) at population densities of 10^1-10^8 CFU plate^{-1} upon exposure to 405-nm light at an irradiance of 0.5 mWcm^{-2} for 16-h (28.8 Jcm^{-2}) and 24-h (43.4 Jcm^{-2}).

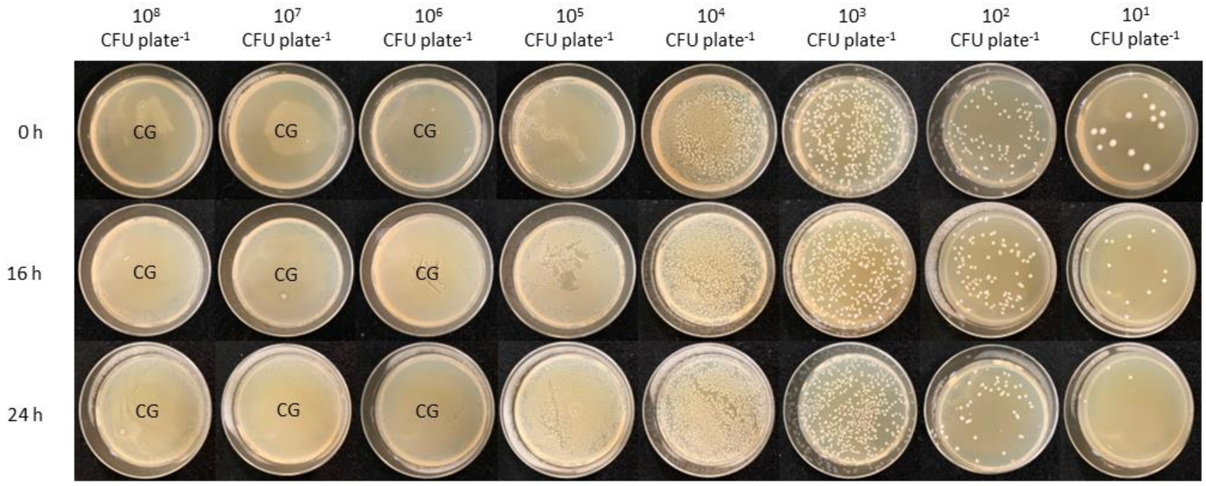

Fig. 1 Appearance of *E. faecium* at 10^8 – 10^1 CFU plate^{-1}, after exposure to 0.5 mWcm-2 405-nm light for 16 h and 24 h (28.8 and 43.4 Jcm-2, respectively). CG represents plates with confluent growth of bacteria.

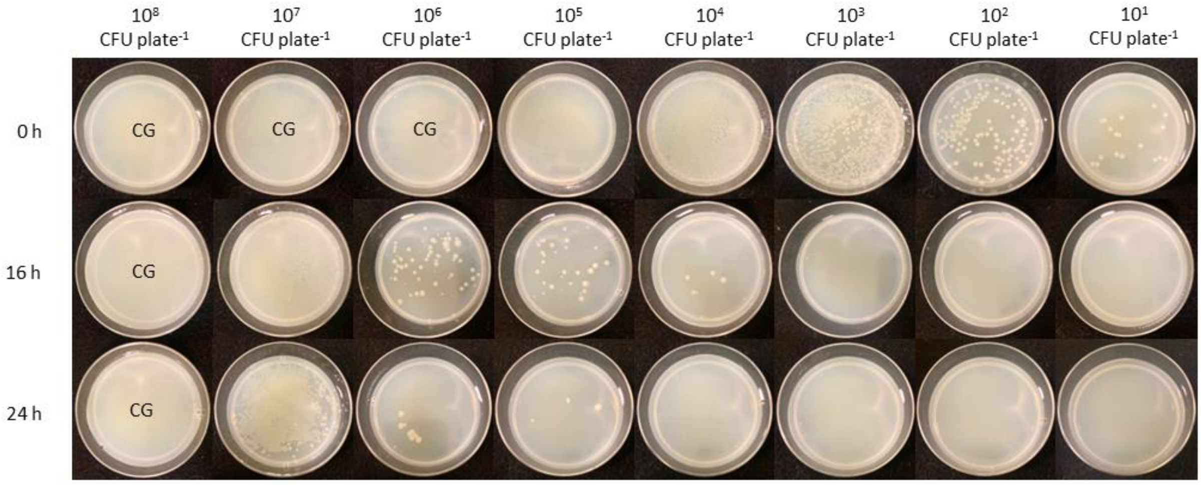

Fig. 2 Appearance of *K. pneumoniae* at 10^8 – 10^1 CFU plate^{-1}, after exposure to 0.5 mWcm-2 405-nm light for 16 h and 24 h (28.8 and 43.4 Jcm-2, respectively). CG represents plates with confluent growth of bacteria.

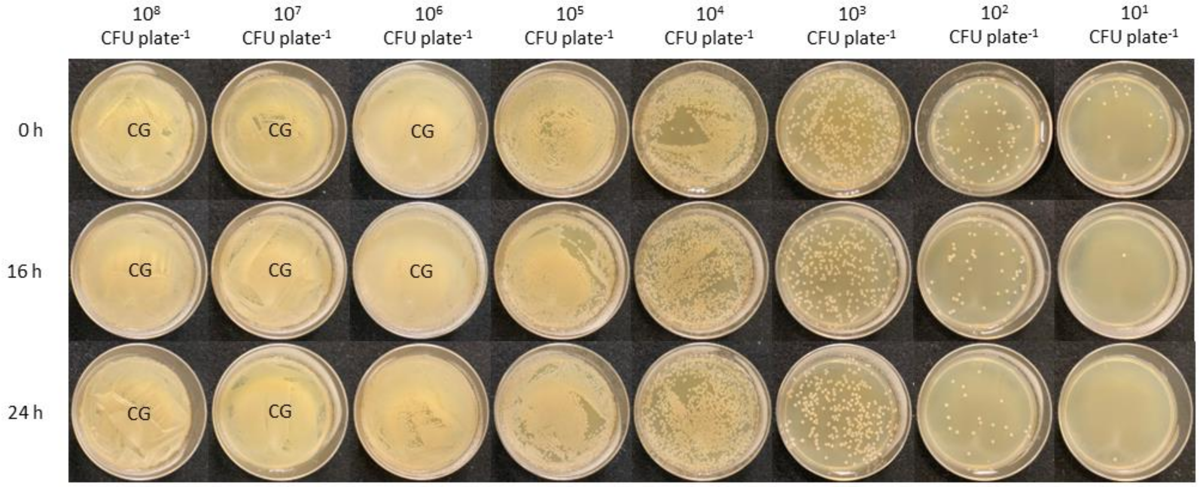

Fig. 3 Appearance of *A. baumannii* at 10^8 – 10^1 CFU plate^{-1}, after exposure to 0.5 mWcm-2 405-nm light for 16 h and 24 h (28.8 and 43.4 Jcm-2, respectively). CG represents plates with confluent growth of bacteria.

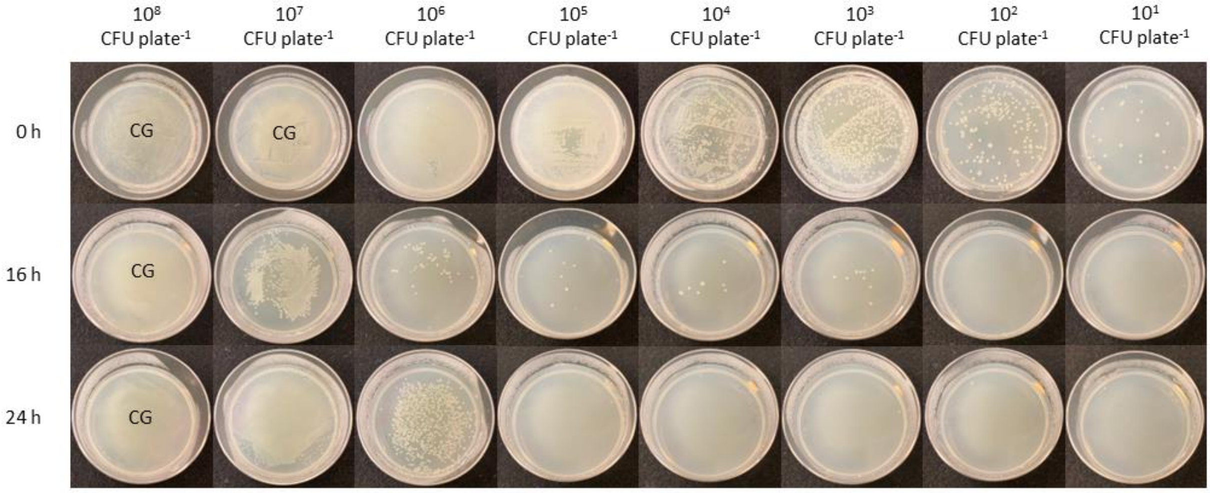

Fig. 4 Appearance of *E. cloacae* at 10^8 – 10^1 CFU plate^{-1}, after exposure to 0.5 mWcm-2 405-nm light for 16 h and 24 h (28.8 and 43.4 Jcm-2, respectively). CG represents plates with confluent growth of bacteria.
